# In-hospital mortality of COVID-19 in Iranian children and youth: A multi-centre retrospective cohort study

**DOI:** 10.7189/jogh.12.05048

**Published:** 2022-11-12

**Authors:** Pedram Fattahi, Sepideh Abdi, Elnaz Saeedi, Samin Sirous, Farnaz Firuzian, Mehdi Mohammadi, Negar Taheri, Mina Khaki, Ali Qandian, Fereshte Lotfi, Arad Iranmehr, Saeed Nemati, Mojtaba Vand Rajabpour

**Affiliations:** 1 Student Research Center, Qazvin University of Medical Sciences, Qazvin, Iran; 2 Cancer Research Center, Cancer Research Institute, Tehran University of Medical Sciences, Tehran, Iran; 3 School of Medicine, Iran University of Medical Sciences, Tehran, Iran; 4 Emergency Medicine Management Research Center, Health Management Research Institute, Iran University of Medical Sciences, Tehran, Iran; 5 Communicable disease office, Deputy of Health, Qazvin University of Medical Sciences, Qazvin, Iran.; 6 Neurological Surgery Department, Imam Khomeini Hospital Complex, Tehran University of Medical Sciences, Tehran, Iran

## Abstract

**Background:**

COVID-19 presents as a mild and less severe respiratory disease among children. However, it is still lethal and could lead to death in paediatric cases. The current study aimed to investigate the clinical characteristics of children and young people hospitalized due to COVID-19 in Qazvin-Iran. We also investigated the risk factors of death due to COVID-19 in paediatric cases.

**Methods:**

We performed a retrospective cohort study on 645 children and young people (ages 0-17) hospitalized since the beginning of the COVID-19 pandemic. The cases were confirmed with positive results of reverse transcription-polymerase chain reaction (RT-PCR). The data were retrieved from an electronic database of demographic, epidemiological, and clinical characteristics.

**Results:**

The median age of the admitted patients was 4.0 years, 33.6% were under 12 months old, and 53.0% were female. Fever, cough, nausea/vomiting, dyspnoea, and myalgia were the most common symptoms presented by 50.5%, 47.6%, 24.2%, and 23.0% of the patients, respectively. Overall, we observed 16 cases of death and the in-hospital fatality rate was 2.5%. We also found comorbidity as an independent risk factor of death (odds ratio (OR) = 3.8, 95% confidence interval (CI) = 1.2-12.1, *P*-value = 0.022). Finally, we observed an increased risk of death in patients with dyspnoea (OR = 11.0, 95% CI = 2.8-43.7).

**Conclusion:**

In-hospital mortality was relatively high in paediatric patients who were hospitalized due to COVID-19 in Iran. The risk of hospitalization, ICU admission, and death was higher among children with younger ages, underlying causes, and dyspnoea.

A respiratory syndrome caused by coronavirus 2019 (SARS-CoV-2) was first introduced in Wuhan, China, in 2019 and soon after became a global pandemic known as COVID-19. COVID-19 is lethal among all age groups. However, it causes less severe diseases among children and young people [1].

The global average age of the COVID-19 infected patients is 47 years, and only 1%-2% of the cases are children and young people [2-4]. Children are less prone to COVID-19 infection, and COVID-19 has led to mild or asymptomatic disease in most reported cases [5,6]. COVID-19 mortality in children is a rare outcome, estimated at least 1% [7]. However, paediatric patients are essential because of more critical paediatric cases due to new variants of the disease, and COVID-19 asymptomatic infection leading to sub-acute and chronic complications among paediatric cases [8,9]. There is also concern regarding increasing Multisystem Inflammatory Syndrome (MIS) among children [10-12], and years of life lost due to COVID-19 mortality in children [13].

COVID-19 mortality in paediatric patients ranged from 0% in Norway to 12% in some reports from Latin America [14-16]. The previous studies in Iran reported paediatric COVID-19 mortality at 5.3% [17] and 8.2% [18] which were higher than the reported values from high-income and developed countries [19,20]. The previous studies addressed younger ages, comorbidity, and dyspnoea as the significant risk factors for COVID-19 mortality [17,18,21]. The difference in characteristics of the hospitalized patients, access to medical care, quality of the provided hospital care, and hospital capacities and infrastructures are the main reasons for such a wide gap in paediatric COVID-19 mortality worldwide [22].

The current study aimed to investigate the in-hospital mortality and the associated risk factors among Children and Young people (ages 0-17) hospitalized with COVID-19 in Qazvin province in Iran.

## METHODS

### Study design and population

The current study was a multicentre retrospective cohort performed on children and young patients with COVID-19 hospitalized in Qazvin province- Iran, over two years of the pandemic, from February 2020 to February 2022. The study was performed on children and young people under the age of 19 with a diagnosis of COVID-19 confirmed with positive results of reverse transcription-polymerase chain reaction (RT-PCR) and hospitalized in one of the hospitals in Qazvin province in Iran for over two years during the COVID-19 pandemic. Early diagnosis was based on nasopharyngeal/oropharyngeal throat swab and lower respiratory secretion specimens (by induced sputum sample or in intubated patients) was performed for definitive diagnosis. The samples were obtained from the patients by a trained technician and sent to SARS-CoV-2 PCR test over their hospitalization.

Case enrolment was performed in 15 hospitals in Qazvin province, covering 1.2 million people and around 378 000 children and youths (Ages 0-17). The data for this study was retrieved from an electronic database provided by Qazvin University of Medical Sciences. This database covers demographic, epidemiological, and clinical characteristics of patients hospitalized due to COVID-19. Please see [23,24] for more detailed information about this database.

### Data collection and outcome

Demographic characteristics (sex, and age), co-existing disorders including cancer, diabetes, liver disorders, COPD and asthma, and immune-deficiency diseases, signs and symptoms (dyspnoea, fever, cough, myalgia, nausea/vomit, headache, diarrhoea, anorexia, dizziness, abdominal pain, seizures, and loss of taste/smell) were collected at the admission time and recorded for each hospitalized patient. We also collected data on the provided treatments (oxygen therapy, ventilation), symptom onset, admission, and discharge date, admission to intensive care unit (ICU), and vital status at discharge time. The primary outcome of the current study were ICU admission, and vital status at discharge.

### Exclusion criteria

Cases aged over 18 were excluded from the study. We also excluded cases that either had a negative result of the RT-PCR test or were not tested. Cases with missing status at discharge were also removed from the study. Patients who were hospitalized for less than 24 hours were also excluded.

### Statistical analysis

We used median and interquartile range (IQR) for continuous variables. Dichotomous variables were shown as frequency (percentage). We used multiple logistic regression to determine the contributing factor of ICU admission and COVID-19 case fatality rate (CFR). For the model generation, we first investigated the association between the baseline characteristics (including: age, sex, comorbidities, clinical signs, and symptoms) and the target outcomes (ICU admission and death) using simple logistic regression. We then used the Wald test to select the most influential variables (with *P* < 0.2) for the multiple logistic regression model. We provided each factor's odds ratio (OR) and 95% confidence interval (CI). All statistical analyses were performed using Stata software (StataCorp. 2014. Stata Statistical Software: Release 14.1, College Station, TX: StataCorp LP), and *P*-values less than 0.05 were considered significant.

## RESULTS

We performed the current study on 645 children and young people hospitalized in the Qazvin province hospitals since the beginning of the COVID-19 pandemic. The median age (IQR) of the admitted patients was 4.0 (15.0) years, where 33.6% of them were under 12 months old, and 53.0% were female. Overall, 35.2% of the patients have been referred to the hospital five days after their symptom onset. The proportion of all types of comorbidity was 10.2%, where diabetes (3.4%), cardiovascular diseases (CVD) (2.0%), and asthma (1.5%) were identified as the most common comorbidities. In 31.4% of the hospitalized patients, oxygen saturation at admission was lower than 93% (**Table 1**).

**Table 1:** Demographic, clinical characteristics, provided treatments, and outcome of patients under 19 hospitalized with confirmed SARS-CoV-2

Variable	Patients (n = 645)
Median age and interquartile range (IQR), years	4.0 (15.0)
Age group (years), n (%)	
<1	217 (33.6%)
1-5	135 (20.9%)
6-12	99 (15.3%)
13-19	194 (30.1%)
Sex at birth, n (%)	
Female	342 (53.0%)
Male	303 (47.0%)
Admission before 5 d of symptom onset, n (%)	
No	374 (58.0%)
Yes	227 (35.2%)
Missing	44 (6.8%)
Any comorbidities, n (%)	
No	579 (89.8%)
Yes	66 (10.2%)
Cancer	4 (0.6%)
Diabetes	22 (3.4%)
Liver disorders	2 (0.3%)
Immunodeficiency	3 (0.5%)
CVD	13 (2.0%)
Asthma/COPD	10 (1.5%)
Sign and symptoms, n (%)	
Fever	326 (50.5)
Cough	307 (47.6)
Dyspnoea	156 (24.2)
Myalgia	148 (23.0)
Nausea/vomiting	96 (14.9)
Headache	75 (11.7)
Diarrhoea	58 (9.0)
Anorexia	51 (7.9)
Seizures	45 (7.0)
Dizziness	24 (3.7)
Abdominal pain	14 (2.2)
Chest pain	14 (2.2)
Loss of smell/taste	14 (2.2)
Oxygen saturation, n (%)	
>93	442 (68.5%)
<93	203 (31.5%)
Supplementary oxygen, n (%)	
No	451 (69.6%)
Yes	194 (30.1%)
Invasive ventilation, n (%)	
No	625 (96.9%)
Yes	20 (3.1%)
ICU admission, n (%)	
No	616 (95.5%)
Yes	29 (4.5%)
Outcome, n (%)	
Recovered	629 (97.5%)
Died	16 (2.5%)

CVD – cardio vascular diseases, COPD – chronic obstructive pulmonary diseases, ICU – intensive care unit

Fever, cough, nausea/vomiting, dyspnoea, and myalgia were the most common symptoms presented by 50.5%, 47.6%, 24.2%, and 23.0% of the patients, respectively (**Table 1**).

Supplementary oxygen was provided for 30.1% of the patients. The overall proportion of the patients with ICU admission was 4.5% (29 patients), and 3.1% of the patients (20 patients) needed invasive mechanical ventilation. The odds of ICU admission were 2.7 times higher in the male gender. Children with comorbidities were also more likely to be admitted to ICU (OR = 2.8, 95% CI = 1.05-7.3, *P* < 0.05). Oxygen saturation at admission was the other influential factor, and patients with PSO_2_<93% had increased odds of admission to critical care (OR = 3.7, 95% CI = 1.6-8.8, *P* < 0.05) (**Table 2**).

**Table 2:** Factors associated with ICU admission and COVID-19 outcome

	ICU admission	Multivariate model	Outcome	Multivariate model
Variable	n (%)	OR (95% CI)	n (%)	OR (95% CI)
Sex				
Female	8 (2.3%)	Ref	9 (2.6%)	Ref
Male	21 (6.9%)	2.7 (1.2-6.5)	7 (2.3%)	0.7 (0.2-1.9)†
Age group				
<1 y	10 (4.6%)	Ref	7 (3.2%)	Ref
1-5 y	7 (5.2%)	1.4 (0.5-4.0)	3 (2.2%)	0.7 (0.1-3.1)
6-12 y	4 (4.0%)	1.2 (0.3-4.1)	2 (2.0%)	0.9 (0.1-4.7)
13-19 y	8 (4.1%)	1.2 (0.4-3.3)	4 (2.0%)	0.8 (0.2-3.2)
Any comorbidities				
No	22 (3.8%)	Ref	10 (1.7%)	Ref
Yes	7 (10.6%)	2.7 (1.05-7.3)	6 (9.1%)	3.8 (1.2-12.1)
Dyspnoea				
No	11 (2.5%)	Ref	3 (0.7%)	Ref
Yes	18 (8.8%)	3.7 (1.6-8.8)*	13 (6.4%)	11.0 (2.8-43.7)*
Fever				
No	12 (3.7%)		6 (1.8%)	
Yes	17 (5.2%)	1.3 (0.6-3.1)*	10 (3.1%)	2.2 (0.7-6.7)*
Cough				
No	20 (5.9%)	Ref	11 (3.2%)	Ref
Yes	9 (2.9%)	0.4 (0.2-1.1)*	5 (1.6%)	0.4 (0.1-1.2)*

ICU – intensive care unit, CI – confidence interval, OR – odds ratio

*Adjusted for age, sex, comorbidity.

†Adjusted for all comorbidities.

### Outcome

Vital status at discharge time was the primary outcome in the current study. Our data showed that, in total, 16 (2.5%) patients died in Qazvin province hospitals during the COVID-19 pandemic. Moreover, having comorbidity was associated with an increased risk of death (OR = 3.8, 95% CI = 1.2-12.1, *P* < 0.05). Patients with dyspnoea had an increased odds of death (OR = 11.0, 95% CI = 2.8-43.7), and the association was statistically significant (*P* < 0.05) (**Table 2**).

## DISCUSSION

The retrospective cohort study was performed on all paediatric COVID-19 patients hospitalized for over two years during the COVID-19 pandemic in Qazvin province-Iran. We aimed to investigate the risk factors of severe COVID-19 and in-hospital mortality in children and youths. The results of our study suggested a 4.5% ICU admission with 2.5% in-hospital fatality rate. Patients with co-existing disorders and dyspnoea had higher risk of in-hospital COVID-19 mortality.

The observed in-hospital case-fatality rate for the paediatric COVID-19 patients admitted in hospitals of Qazvin province was considerably fewer than the previous reports in Iran (5.3%) (17) as well as the reported results by the Latin American countries (8.9%) [25]. Differences in case definition and characteristics of the admitted patients, like a higher proportion of patients with underlying causes or younger age, were the main drivers of such differences in the case fatality rate. For instance, Madani et al. reported higher proportion of sever cases with dyspnoea [17]. However, the fatality rate in our study was still tremendous when compared with high-income and developed countries such as the USA [20], Norway [14], and Italy [19], where in-hospital case fatality rate of COVID-19 was less than 0.1%. The main reasons for such differences are better access to medical care, higher awareness of infectious diseases, higher socioeconomic status, and high-quality services in high-income countries [22]. Moreover, comparison of Iranian paediatric patients with other counties [17,18], showed higher prevalence of dyspnoea among Iranian children with COVID-19. Delay in receiving medical care, hospitalization, and high prevalence of vitamin D deficiency are the main reasons that have already been discussed to justify such circumstances and the high proportion of dyspnoea in COVID-19 cases in Iranian children [17]. This high prevalence could be considered as one reason for such a high case fatality in Iranian paediatric patients compared to the high-income countries.

Children younger than one year were the most frequently admitted patients in our study that was consistent with previous reports suggesting that infants are more at risk of hospitalization due to COVID-19 among paediatric patients [17]. Higher sensitivity to fever and diarrhoea plus incomplete vaccination were argued as the main drivers of severe disease in this age group [17]. The highest in-hospital mortality was also observed in patients younger than 1 year, however, there was no association between age and COVID-19 complications such as ICU admission and COVID-19 mortality. Our findings were in contrast of the previous reports have been conducted in the USA [26], China [27], and Iran [17], in which infants were identified as the most at-risk group for ICU admission and death due to COVID-19. Lower sample size and lack of statistical power to investigate such association might be the main reason of this difference.

Fever, cough, and dyspnoea were the most prevalent symptoms of the SARS CoV-2 among the hospitalized children in this study, and our findings were supported by the previous studies conducted on children or adults [17,28,29]. Our study found dyspnoea as the only symptom associated with a higher risk of death. In more detail, our results suggested that the odds of death in children with dyspnoea were 3.6 times higher than the ones without this symptom. This finding was consistent with the results reported by the previous studies [17].

Pre-existing disorders were observed among 10.2% of the hospitalized children. This finding was consistent with the results of the previous study conducted in Iran [17]; however, it was much lower than the reported findings by Armin et al. [18]. Comorbidities were a decisive risk factor for ICU admission and COVID-19 mortality. According to our findings the odds of ICU admission among children with at least one comorbidity were 2.7 times higher compared with the other patients. Moreover, these patients had an increased risk of in-hospital mortality than the other patients. These findings were consistent with the results of the previously conducted studies [17,18,30,31].

### Strengths and limitations

Our data was unique as it provides information on the confirmed paediatric patients hospitalized with COVID-19 over 2-year pandemic in Iran. Moreover, we included all confirmed cases and did not restrict our analysis to only paediatric hospitals. However, the data had some limitations and should be interpreted in light of these limitations. Our COVID-19 registry in Iran did not cover laboratory data, and there was no data on pharmacological treatments. Moreover, we only included patients with confirmed PCR tests, while the test's specificity was not 100% even in the best situation. Therefore, there might be some false negatives that we removed.

## CONCLUSION

COVID-19 has led to relatively high in-hospital mortality in paediatric patients in Iran. Children with younger ages, underlying causes, and dyspnoea were the high more at-risk groups that should have been given priority for hospitalization and admission to ICU.
